# Nonalcoholic Fatty Liver Disease Is Independently Associated with Early Left Ventricular Diastolic Dysfunction in Patients with Type 2 Diabetes

**DOI:** 10.1371/journal.pone.0135329

**Published:** 2015-08-07

**Authors:** Alessandro Mantovani, Matteo Pernigo, Corinna Bergamini, Stefano Bonapace, Paola Lipari, Isabella Pichiri, Lorenzo Bertolini, Filippo Valbusa, Enrico Barbieri, Giacomo Zoppini, Enzo Bonora, Giovanni Targher

**Affiliations:** 1 Section of Endocrinology, Diabetes and Metabolism, Department of Medicine, University and Azienda Ospedaliera Universitaria Integrata of Verona, Verona, Italy; 2 Section of Cardiology, Department of Medicine, University and Azienda Ospedaliera Universitaria Integrata of Verona, Verona, Italy; 3 Division of Cardiology, ‘‘Sacro Cuore” Hospital, Negrar (VR), Italy; 4 Division of General Medicine and Diabetes Unit ‘‘Sacro Cuore” Hospital, Negrar (VR), Italy; Temple University, UNITED STATES

## Abstract

Accumulating evidence suggests that nonalcoholic fatty liver disease (NAFLD) is associated with left ventricular diastolic dysfunction (LVDD) in nondiabetic individuals. To date, there are very limited data on this topic in patients with type 2 diabetes and it remains uncertain whether NAFLD is independently associated with the presence of LVDD in this patient population. We performed a liver ultrasonography and trans-thoracic echocardiography (with speckle-tracking strain analysis) in 222 (156 men and 66 women) consecutive type 2 diabetic outpatients with no previous history of ischemic heart disease, chronic heart failure, valvular diseases and known hepatic diseases. Binary logistic regression analysis was used to examine the association between NAFLD and the presence/severity of LVDD graded according to the current criteria of the American Society of Echocardiography, and to identify the variables that were independently associated with LVDD, which was included as the dependent variable. Patients with ultrasound-diagnosed NAFLD (*n* = 158; 71.2% of total) were more likely to be female, overweight/obese, and had longer diabetes duration, higher hemoglobin A1c and lower estimated glomerular filtration rate (eGFR) than those without NAFLD. Notably, they also had a remarkably greater prevalence of mild and/or moderate LVDD compared with those without NAFLD (71% vs. 33%; P<0.001). Age, hypertension, smoking, medication use, E/A ratio, LV volumes and mass were comparable between the two groups of patients. NAFLD was associated with a three-fold increased odds of mild and/or moderate LVDD after adjusting for age, sex, body mass index, hypertension, diabetes duration, hemoglobin A1c, eGFR, LV mass index and ejection fraction (adjusted-odds ratio 3.08, 95%CI 1.5–6.4, *P* = 0.003). In conclusion, NAFLD is independently associated with early LVDD in type 2 diabetic patients with preserved systolic function.

## Introduction

Non-alcoholic fatty liver disease (NAFLD) is increasingly diagnosed worldwide and is the most common chronic liver disease in patients with type 2 diabetes (occurring in up to 70–75% of these patients) [1–3]. In addition, patients with type 2 diabetes are more likely to develop the more severe forms of NAFLD, such as non-alcoholic steatohepatitis (NASH), advanced fibrosis and cirrhosis [1,4,5]. This finding may help explain epidemiological studies from populations across the world that have reported a twofold to fourfold increase in risk of developing cirrhosis, liver failure and hepatocellular carcinoma in people with type 2 diabetes [1,6].

To date, there is ample evidence suggesting that NAFLD is associated not only with liver-related morbidity and mortality, but also with an increased risk of developing cardiovascular disease, which is the most common cause of death among patients with type 2 diabetes [6–8]. In recent years, accumulating evidence also indicates that in non-diabetic individuals, the presence of NAFLD is strongly associated with cardiac dysfunction, mainly left ventricular diastolic dysfunction (LVDD), as well as with an increased risk of cardiac arrhythmias, mainly atrial fibrillation [9].

To our knowledge, very little information is currently available regarding the relationship between NAFLD and LVDD in people with type 2 diabetes, a group of individuals in whom structural and functional myocardial abnormalities may develop even in the absence of hypertension or ischemic heart disease (IHD) [10]. In a small study, involving 50 type 2 diabetic patients without a prior history of IHD, we recently reported a significant association between NAFLD and early LVDD (defined as altered E/e’ ratio), which was independent of age, sex, hemoglobin A1c, and hypertension status [11]. However, given the relatively small sample size of this study, we were unable to perform a more extensive adjustment for other potential confounders [11].

Thus, the aim of the present study was to examine whether NAFLD is associated with LVDD (as detected by spectral tissue Doppler imaging, which is currently the most reliable diagnostic approach for evaluating subclinical myocardial function abnormalities) [10,12] in a large sample of patients with type 2 diabetes, and to determine to the extent to which diabetes-related variables and echocardiographic parameters can mediate this association.

## Materials and Methods

We studied 222 white consecutive outpatients with type 2 diabetes, who regularly attended our diabetes clinics of the University of Verona and the “Sacro Cuore” Hospital of Negrar. For the current study, we excluded patients with: (1) a prior history of IHD (*i*.*e*., myocardial infarction, angina or coronary revascularization procedures), chronic heart failure, valvular diseases, atrial fibrillation, malignancy and end-stage renal disease; and (2) a prior history of cirrhosis of any etiology or other known causes of chronic liver diseases, including viral hepatitis, hemochromatosis and excessive alcohol intake (defined as >30 g/day of alcohol for men and >20 g/day for women, respectively). All women were postmenopausal and did not take hormonal replacement therapy.

The local Ethics Committee (University of Verona) approved the study protocol. All participants gave their written informed consent for participation in this medical research.

Body mass index (BMI) was calculated by dividing weight in kilograms by height in meters squared. A physician measured blood pressure with a mercury sphygmomanometer (at the right upper arm using an appropriate cuff size) after patients had been seated quietly for at least 5 minutes. Patients were considered to have hypertension if their blood pressure was ≥140/90 mmHg or if they were taking any anti-hypertensive drugs. Pulse pressure was determined as the difference between the systolic pressure and diastolic pressure. Information on alcohol consumption, smoking history and use of medications was obtained from all patients via interviews during medical examinations.

Venous blood samples were drawn in the morning after an overnight fast. Serum liver enzymes, creatinine (measured using a Jaffé rate-blanked and compensated assay) and other biochemical blood measurements were determined using standard laboratory procedures (DAX 96; Bayer Diagnostics, Milan, Italy). Normal ranges for serum aspartate and alanine aminotransferases and gamma-glutamyltransferase in our laboratory were 10–40 U/l for women and 10–50 U/l for men, respectively. LDL-cholesterol was calculated using the Friedewald’s formula. Hemoglobin A1c (HbA1c) was measured by an automated high-performance liquid chromatography analyzer (HA-8140; Menarini Diagnostics, Florence, Italy); the upper limit of normal for our laboratory was 5.6%. Glomerular filtration rate (eGFR) was estimated by the 4-variable Modification of Diet in Renal Disease study equation [13]. Albuminuria was measured by an immuno-nephelometric method on a morning spot urine sample and expressed as the albumin-to-creatinine ratio; abnormal albuminuria was defined as an albumin-to-creatinine ratio ≥30 mg/g creatinine.

Presence of either internal or common carotid artery stenoses was ascertained by echo-Doppler scanning. In all participants, the presence of microvascular diabetic complications, such as peripheral sensory neuropathy (by biothesiometer), nephropathy (by eGFR and albuminuria measurements) and retinopathy (by fundoscopy after pupillary dilation) was also recorded.

Liver ultrasonography was performed in all participants by two experienced and trained radiologists, who were blinded to the subjects’ details, including echocardiographic data. Hepatic steatosis was diagnosed on the basis of characteristic ultrasonographic features, *i*.*e*., evidence of diffuse hyperechogenicity of the liver relative to the kidneys, ultrasound beam attenuation and poor visualization of intrahepatic vessel borders and diaphragm [14]. It is known that ultrasonography has good sensitivity and specificity for detecting moderate and severe hepatic steatosis (90–95%), but its sensitivity is reduced when the hepatic fat infiltration upon liver biopsy is <30% [14]. No information was available on estimates of intra- or inter-rater reliability of liver ultrasonography. In addition, semi-quantitative ultrasonographic scoring for the degree of hepatic steatosis (mild, moderate or severe) was not also available in this study. Grading of hepatic fat content using ultrasonography has been used in previous studies but remains somewhat subjective [14].

A 12-lead standard resting electrocardiogram and a transthoracic echocardiographic Doppler evaluation with spectral tissue Doppler analysis (Vivid 7, GE Vingmed, Horten, Norway) were performed within approximately 1 month of liver ultrasonography in all patients by two experienced and trained cardiologists, who were blinded to the participants’ details, including liver ultrasound data. Conventional echocardiography was used to measure left ventricular (LV) diameters, wall thickness, and mass according to international standard criteria [15]. LV end-diastolic (EDV) and end-systolic volumes and ejection fraction (EF) at rest were measured at the apical 4-chamber and 2-chamber views (by modified Simpson rule) [15]. Left atrial maximal volume was measured at the end of LV systole from the apical 4-chamber and 2-chamber views (by modified Simpson rule) [15]. Measurements were indexed to body surface area when appropriate. Pulsed-wave Doppler was used to measure trans-mitral peak early diastolic velocity (E), peak late diastolic velocity (A) and E-wave deceleration time (Dte). Isovolumetric relaxation time (IVRT) was also calculated [16]. Each value was obtained from the average of three measurements. Systemic arterial compliance (SAC) was estimated by the stroke volume–to–pulse-pressure ratio and systemic vascular resistance (SVR) index by mean arterial pressure ÷ cardiac index × 80 [15,16].

Pulsed-wave tissue Doppler echocardiography of the septal and lateral mitral annulus was used to measure the early (e’) and late (a’) annular diastolic and systolic (s’) tissue velocities, and the mean values of septal and lateral annulus measurements were used for analysis [17–19]. The e’ tissue velocity is relatively preload independent and correlates inversely with the time constant for isovolumic relaxation (*tau*), which is derived from the following formula: *tau* = (14.70–100 x e’)/0.15 [20]. LV end-diastolic pressure (EDP) was estimated as follows: EDP = 11.96 + 0.596 x E/e’ ratio [20].

Myocardial deformation measurements were also performed off-line (by the same cardiologist who performed echocardiographic examinations) in a subgroup of patients with adequate apical windows with the use of a standard EchoPac PC workstation application (GE Healthcare, Wisconsin, USA) for 2-dimensional speckle-tracking myocardial strain analysis. Global longitudinal strain and strain rate curves were obtained in 156 patients, including all six LV myocardial segments from 4-chamber, 2-chamber, and long-axis apical views [21–23]. The average values of peak systolic longitudinal strain and peak systolic strain rate from the 3-apical views were calculated as global longitudinal strain (LS_SYS_) and global strain rate (SR_SYS_), respectively. Similarly, the global strain rate during the early (SR_E_) and late (SR_L_) phase of diastole was also calculated. The ratio of trans-mitral E-wave velocity to SR_E_ as an index of LV filling pressure was calculated as previously proposed [19]. Standard echocardiographic views were obtained using frequency, depth, and sector width adjusted for frame-rate optimization (between 60 and 100 fps). In a previous study [23], we have shown that when tissue Doppler imaging signals were re-measured by the same observer the mean absolute differences (±SD) in tissue velocities within the same observer were 0.10±0.02 cm/s for s’ velocity, 0.19±0.17 cm/s for e’ velocity, and 0.23±0.20 cm/s for a’ velocity, respectively (*P* = NS for all differences). Similarly, when tissue Doppler imaging signals were re-measured by a second observer, the mean absolute differences in tissue velocities between the 2 observers were 0.11±0.09 cm/s for s’ velocity, 0.30±0.25 cm/s for e’ velocity, and 0.36±0.28 cm/s for a’ velocity (*P* = NS for all differences). No significant differences were also found in the intra-observer and inter-observer variabilities for global longitudinal strain, SR_SYS_, and SR_E_ [23].

In all participants LV diastolic function was categorized as normal, mild, moderate or severe dysfunction using the echocardiographic criteria that have been proposed and validated by the American Society of Echocardiography (ASE). The individual criteria in the ASE algorithm used to grade LV diastolic function were mitral E/A ratio, Dte, e’ septal, e’ lateral, averaged E/e’ ratio, indexed left atrial volume, and mitral (A) and pulmonary vein (Ar)-wave durations [16,24].

## Statistical Analysis

Data are presented as means±SD, medians and inter-quartile ranges (IQR) or percentages. Differences in main clinical and biochemical characteristics and echocardiographic parameters among patients stratified by NAFLD status were tested with the unpaired Student’s *t*-test for normally distributed variables and the Mann-Whitney test for non-normally distributed variables; the X^2^ test with Yates’s correction for continuity was used to test differences in categorical variables between the groups. Binary logistic regression analysis was used to examine the association between NAFLD and the presence/severity of LVDD graded according to current ASE criteria (absent *vs*. mild and moderate grades combined; in our study no patients had severe LVDD as specified below), and to identify the variables that are independently associated with LVDD, which was included as the dependent variable. Four forced-entry multivariable logistic regression models were performed: an unadjusted model; a model adjusted for age and sex (model 1); a regression model further adjusted for BMI, diabetes duration, HbA1c, eGFR and hypertension (model 2); and, finally, a model adjusted for the same variables included in model 2 *plus* echocardiographic parameters, such as LV ejection fraction and indexed LV mass (model 3). The covariates for multivariable regression analyses were chosen as potential confounding factors based on their significance in univariate analyses or based on their biological plausibility (*i*.*e*., hypertension and indexed LV mass). Interaction terms were also generated between NAFLD and sex, age and BMI in terms of LVDD. None of these interaction terms was statistically significant in the fully adjusted regression models. *P*-values <0.05 were considered statistically significant.

## Results

Of the 222 patients (156 men and 66 women) included in the study, 158 (71.2%) patients met the clinical criteria for a diagnosis of NAFLD (*i*.*e*., hepatic steatosis on ultrasonography among patients who did not have excessive alcohol consumption, viral hepatitis, drug-induced liver disease or other known causes of liver disease) and 64 patients did not. Overall, 91 patients had normal diastolic function, whereas the remaining 131 (59%) patients had mild or moderate grades of LVDD; no patients had severe LVDD. No patients had clinical, biochemical characteristics (including platelet count, serum liver enzymes, albumin and prothrombin time) or ultrasonographic findings suggestive of cirrhosis or portal hypertension (coarse liver texture or splenomegaly).

**Table 1**shows the main clinical and biochemical characteristics of patients stratified by NAFLD status. Patients with NAFLD were more likely to be female (36.1% *vs*. 14.7%), overweight/obese, and had a longer duration of diabetes compared with those without NAFLD. They also had significantly higher values of HbA1c and serum gamma-glutamyltransferase, had a greater prevalence of chronic kidney disease (*i*.*e*., eGFR <60 ml/min/1.73 m^2^), and tended to have higher blood pressure values and to receive more anti-hypertensive medications. The two groups did not differ significantly in terms of age, smoking, pulse pressure, mean arterial pressure, heart rate, fasting glucose levels, lipids, serum aminotransferases, abnormal albuminuria, diabetic retinopathy, peripheral sensory neuropathy, carotid artery stenoses>50%, and current use of hypoglycemic, lipid-lowering and anti-hypertensive drugs (including beta-blockers and renin-angiotensin-aldosterone system inhibitors). No patients were taking pioglitazone or glucagon-like peptide-1 receptor agonists.

**Table 1:** Clinical and biochemical characteristics of patients with type 2 diabetes stratified by presence or absence of NAFLD.

	Without NAFLD (*n* = 64)	With NAFLD (*n* = 158)	*P* value
Sex (male/female)	55/9	101/57	<0.01
Age (years)	66.9 ± 7	68.6 ± 7	0.11
Weight (kg)	81.2 ± 10	82.1 ± 15	0.64
Body mass index (kg/m^2^)	27.4 ± 3	29.3 ± 5	<0.005
Diabetes duration (years)	9 (5–15)	13 (7–20)	<0.05
Systolic blood pressure (mmHg)	139.7 ± 15	143.9 ± 16	0.07
Diastolic blood pressure (mmHg)	78.0 ± 9	79.4 ± 9	0.26
Pulse pressure (mmHg)	61.7 ± 14	64.5 ± 14	0.19
Mean arterial pressure (mmHg)	98.8 ± 9	99.8 ± 12	0.51
Heart rate (bpm)	74.6 ± 10	74.1 ± 11	0.77
Smoking history (%)	50.0	34.5	0.09
Fasting glucose (mmol/l)	8.0 ± 2.5	8.6 ± 2.1	0.73
Hemoglobin A1c (%)	6.9 ± 0.9	7.4 ± 1.3	<0.005
Total cholesterol (mmol/l)	4.39 ± 0.9	4.45 ± 0.9	0.58
HDL cholesterol (mmol/l)	1.25 ± 0.3	1.27 ± 0.3	0.58
LDL cholesterol (mmol/l)	2.56 ± 0.9	2.54 ± 0.8	0.85
Triglycerides (mmol/l)	1.34 (0.9–1.9)	1.36 (1.1–2.0)	0.49
AST (U/l)	20 (7–36)	23 (8–38)	0.53
ALT (U/l)	23 (9–37)	27 (7–40)	0.21
GGT (U/l)	19 (9–54)	34 (11–65)	<0.05
Hypertension (%)	73.4	81.6	0.08
eGFR <60 ml/min/1.73 m^2^ (%)	3.1	15.2	<0.01
Abnormal albuminuria (%)	18.4	25.7	0.20
Diabetic retinopathy, any degree (%)	6.3	18.9	0.07
Diabetic sensory neuropathy (%)	9.4	16.5	0.16
Carotid artery stenosis ≥50% (%)	9.4	20.3	0.15
Oral hypoglycemic drug users (%)	70.3	81.6	0.08
Insulin users (%)	40.6	35.8	0.42
ACE-inhibitors/ARB users (%)	67.2	77.0	0.33
Calcium-channel blocker users (%)	23.4	33.5	0.14
Diuretic users (%)	26.6	39.0	0.10
Beta-blocker users (%)	7.8	21.5	0.08
Statin users (%)	79.7	74.1	0.37

Sample size, *n* = 222. Data are expressed as means ± SD, medians and interquartile range (IQR) or percentages.

Differences were tested by the chi-squared test for categorical variables, the unpaired Student’s t-test for normally distributed continuous variables and the Mann-Whitney test for non-normally distributed continuous variables (i.e., duration of diabetes, triglycerides and liver enzymes).

Hypertension was defined as blood pressure ≥140/90 mmHg and/or use of any antihypertensive drugs. ALT, alanine aminotransferase; AST, aspartate aminotransferase; ARB, angiotensin II receptor blockers; eGFR, estimated glomerular filtration rate; GGT, gamma-glutamyltransferase.

**Table 2**shows the echocardiographic characteristics of patients grouped according to NAFLD status. Compared with those without NAFLD, patients with NAFLD had echocardiographic features of early LVDD as detected by tissue Doppler imaging, *i*.*e*., lower e’ tissue velocity, higher E/e’ ratio, higher Tau, higher LV-EDP, higher EDP/EDV ratio. They also had a slightly larger left atrial volume and lower LV-ejection fraction. Again, when we performed global strain and strain rate measurements in a subgroup of these patients (*n* = 156), we confirmed that NAFLD patients had a significantly higher E/SR_E_ ratio than those without NAFLD. As also shown in **Table 2**, no significant differences were found in LV volumes, E and A trans-mitral wave velocities, E/A ratio, Dte, s’ velocity, a’ velocity, IVRT, Ar-A duration, SVR index and SAC between the two groups of patients.

**Table 2:** Main echocardiographic characteristics of patients with type 2 diabetes stratified by presence or absence of NAFLD.

	Without NAFLD (*n* = 64)	With NAFLD (*n* = 158)	*P* value
LV end-diastolic volume (ml)	106.4 ± 20	105.4 ± 25	0.79
LV end-systolic volume (ml)	37.5 ± 13	39.8 ± 13	0.25
LV ejection fraction (%)	65.4 ± 7	62.8 ± 6	<0.05
LV mass index (g/m^2^)	103.7 ± 20	106.7 ± 25	0.39
Left atrial volume index (ml/m^2^)	28.8 ± 8	31.7 ± 9	<0.05
E wave (cm/s)	62.7 ± 17	66.9 ± 15	0.10
A wave (cm/s)	79.2 ± 14	81.6 ± 33	0.58
E/A ratio	0.78 ± 0.2	0.74 ± 0.2	0.11
Dte (ms)	261.4 ± 61	250.4 ± 66	0.28
s’ velocity (cm/s)	10.1 ± 2.0	12.7 ± 3.9	0.66
a’ velocity (cm/s)	12.0 ± 2.1	11.6 ± 2.5	0.23
e’ velocity (cm/s)	9.3 ± 1.9	7.6 ± 1.7	<0.001
E/e’ ratio	6.9 ± 2.0	9.6 ± 2.0	<0.001
IVRT (ms)	83.8 ± 13	86.5 ± 16	0.26
Tau (ms)	37.5 ± 12	51.3 ± 12	<0.001
Ar-A (ms)	6.6 ± 34	16.3 ± 40	0.12
LV-EDP (mmHg)	15.9 ± 1.5	17.7 ± 1.8	<0.001
LV-EDP/EDV ratio (mmHg/ml)	0.16 ± 0.03	0.18 ± 0.05	<0.005
SAC (mmHg/ml)	1.16 ± 0.3	1.11 ± 0.3	0.20
SVR index (dyne/s/cm^5^)	2428 ± 721	2607 ± 851	0.16
LS_SYS_ (%)	-16.2 ± 2.3	-15.9 ± 3.0	0.64
SR_SYS_ (s^-1^)	-1.05 ± 0.15	-1.02 ± 0.25	0.47
SR_E_ (s^-1^)	1.14 ± 0.26	1.05 ± 0.27	0.10
SR_L_ (s^-1^)	1.08 ± 0.23	1.16 ± 0.36	0.20
E/SR_E_ ratio (m)	0.55 ± 0.20	0.68 ± 0.24	<0.005

Sample size, *n* = 222 (except for LV global longitudinal strain and strain rate measurements that were available in 156 patients). Data are means ± SD. Differences were tested by the unpaired Student’s t-test.

EDP, end-diastolic pressure; EDV, end-diastolic volume; IVRT, iso-volumetric relaxation time; LS_SYS_, global longitudinal strain; LV, left ventricular; SAC, systemic arterial compliance; SR_SYS_, global strain rate; SR_E_, global diastolic strain rate during early phase of diastole; SR_L_, global diastolic strain rate during late phase of diastole; SVR, systemic vascular resistance; Tau, time constant of isovolumic relaxation.

After stratifying participants by sex, both female patients with NAFLD (*n* = 57) and male patients with NAFLD (*n* = 101) had lower e’ tissue velocity (7.8±1.7 *vs*. 9.4±2.0 cm/s, *P*<0.001 for men, and 7.7±1.7 *vs*. 9.1±2.0 cm/s, *P* = 0.059 for women, respectively) and higher E/e’ ratio (9.4±2.4 *vs*. 6.9±2.1, *P*<0.001 for men, and 10.1±2.6 *vs*. 6.8±1.4, *P*<0.001 for women, respectively) compared with female (*n* = 9) and male (*n* = 55) patients without NAFLD.

**Fig 1:**
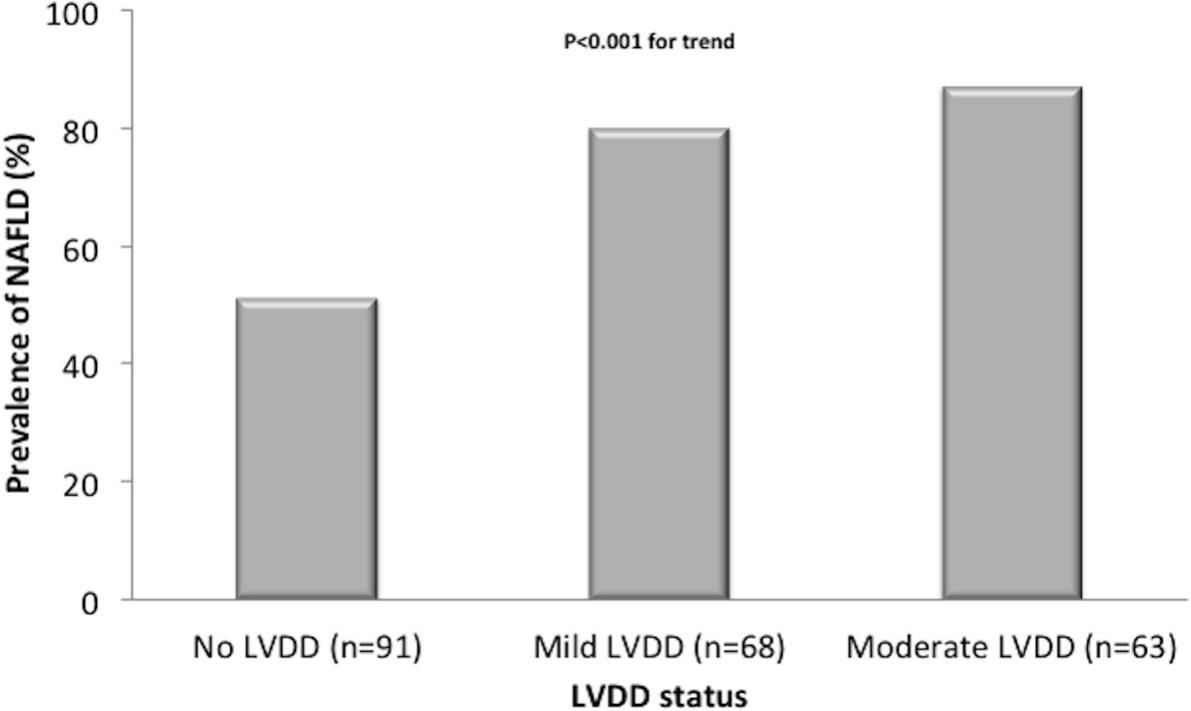
Prevalence of NAFLD in relation to echocardiographic grades of left ventricular diastolic dysfunction (LVDD) in patients with type 2 diabetes. *P*-value <0.001 for the trend among the three groups of patients (as assessed by the χ^**2**^ test).

Notably, as shown in **Fig 1**, there was a strong, graded relationship between NAFLD and the echocardiographic severity of LVDD after stratifying all patients by the current ASE criteria (P-value <0.001 for the trend among the three groups, as assessed by the χ2 test).

**Table 3**shows the effect of the adjustment for several potential confounders on the relationship between NAFLD and presence of mild and/or moderate LVDD. In univariable logistic regression analysis, NAFLD was associated with a ~five-fold increased odds of mild and/or moderate LVDD (unadjusted odds ratio 4.89, 95% CI 2.6–9.2). When we analyzed men and women separately, the presence of NAFLD was associated with an increased odds of LVDD in both sexes: unadjusted odds ratio of 5.13, 95%CI 2.5–10.6 for men and unadjusted odds ratio of 2.47, 95%CI 0.7–10.4 for women, respectively (with a borderline significance for women, possibly due to the relatively small number of women without NAFLD). After adjustment for age and sex (model 1), NAFLD maintained a significant association with LVDD. Further adjustments for BMI, duration of diabetes, HbA1c, eGFR and hypertension (defined as blood pressure ≥140/90 mmHg or use of any anti-hypertensive medication) (model 2) did not appreciably weaken this association. Additional inclusion in this regression model of relevant echocardiographic parameters, such as LV-EF and indexed LV mass (model 3), did not weaken the strong association between NAFLD and LVDD. Of note, other independent predictors of LVDD were a longer duration of diabetes, male sex and lower LV-EF.

**Table 3:** Independent predictors of the presence of mild and/or moderate LV diastolic dysfunction in patients with type 2 diabetes.

Logistic Regression Models	Odds ratio	95% CI	*P* value
**NAFLD (yes *vs*. no)**			
Unadjusted model	4.89	2.6–9.2	<0.001
Adjusted model 1	4.17	2.1–8.1	<0.001
Adjusted model 2	3.50	1.7–7.2	<0.001
Adjusted model 3	3.08	1.5–6.4	= 0.003
**Other independent predictors of LVDD in adjusted model 3**			
Diabetes duration (years)	1.07	1.03–1.1	<0.005
Male sex	3.21	1.4–7.3	<0.005
LV ejection fraction (%)	0.91	0.86–0.96	<0.001

Sample size, *n* = 222. Data are expressed as odds ratios ± 95% confidence intervals (CI) as assessed by either univariable (unadjusted) or multivariable logistic regression analyses. Presence of mild and/or moderate LVDD, *i*.*e*., the dependent variable, was based on criteria proposed by the American Society Echocardiography.Other covariates included in multivariable regression models, along with NAFLD, were as follows: **model 1**: age and sex; **model 2**: age, sex, BMI, duration of diabetes, hemoglobin A1c, eGFR and hypertension (*i*.*e*., blood pressure ≥140/90 mmHg and/or on drug treatment); **model 3**: adjustment for the same variables included in model 2 *plus* LV ejection fraction and LV mass index.

## Discussion

To date, the data available regarding the association between NAFLD and LVDD in patients with type 2 diabetes mellitus are scarce. To our knowledge, the only study available in the literature is that published by our group on a small sample of 50 outpatients with type 2 diabetes, in which we had reported a positive association between NAFLD and early LVDD, defined as increased E/e’ ratio (*i*.*e*., an index of LV filling pressure) [11]. In that study, we found that this association was independent of age, sex, hypertension, and diabetes-related variables. However, given the relatively small sample size of that study (n = 50), we were unable to fully adjust for other potential confounders, such as kidney function and echocardiographic parameters. Additionally, it is important to note that the diagnosis of LVDD using only an altered E/e’ ratio and not accounting for other echocardiographic parameters could be challenged [16,24].

To our knowledge, this is the largest cross-sectional study aimed at examining the association between NAFLD and LVDD in an outpatient sample of type 2 diabetic individuals. In the present study, we confirm and extend the results of our previously published study showing that there was a strong, graded relationship between NAFLD and the severity of LVDD (according to the ASE diagnostic criteria). None of these participants were included in our previously published study. Notably, the association between NAFLD and LVDD remained statistically significant even after adjusting for a larger number of potential confounders, including diabetes-related variables, kidney function parameters, hypertension, and relevant echocardiographic parameters. Moreover, it is also important to note that the use of hypoglycemic, lipid-lowering and anti-hypertensive drugs was not significantly different between patients with and without NAFLD. Again, no patients were treated with pioglitazone or glucagon-like peptide-1 receptor agonists, which have been shown to reduce hepatic steatosis [1,9,25] and improve cardiac function [26,27]. Finally, our novel finding of a significant association between NAFLD and larger left atrial volume might also be of pathophysiological relevance in the explanation of recent observations documenting that type 2 diabetic patients with NAFLD are at risk of atrial fibrillation [28,29].

Collectively, our results suggest that NAFLD may contribute to impairments of both active and passive LV diastolic properties that are most probably additive to the myocardial defects already present in type 2 diabetes. Since no significant differences in afterload conditions in terms of either “steady” or “pulsatile” components of the arterial load (*i*.*e*., mean arterial pressure, SVR index, pulse pressure and SAC) were found between patients with and without NAFLD, our findings also suggest that the observed abnormalities in LV diastolic function could be not attributable to different LV afterload conditions. However, we assessed afterload only at rest and, therefore, we cannot exclude that the hemodynamic response to exercise might differ significantly between the two groups of patients despite similar resting conditions [30].

The putative pathophysiological mechanisms linking NAFLD to LVDD are not fully understood. To date, it remains debatable whether NAFLD is simply a marker of co-existing cardio-metabolic risk factors and different ectopic fat depots (such as visceral adipose tissue, myocardial and pericardial fat) in people at increased risk for cardiac abnormalities, or is an independent risk factor for the development and progression of cardiac function abnormalities.

To date, a number of case-control studies have demonstrated that nondiabetic patients with NAFLD, both adults and children/adolescents, have echocardiographic features of early LVDD compared with their counterparts without NAFLD [9,31–37]. These myocardial functional abnormalities appear to be independent of multiple cardio-metabolic risk factors. Notably, in a study involving 108 overweight or obese children, Pacifico *et al*. [36] also reported a positive, graded relationship between the severity of NAFLD histology and some features of early LVDD. A multiple logistic regression analysis revealed that NAFLD was the only statistically significant variable associated with increased E/e' ratio [36].

In respect to mechanisms how NAFLD might adversely impact on cardiac function and structure, there is now increasing evidence suggesting that NAFLD is not simply an epiphenomenon of structural and functional myocardial abnormalities, but may also contribute to their pathogenesis [8,9]. According to the lipotoxicity theory [38,39], it is likely that there is a pathogenic ‘cross-talk’ between the liver and the expanded and inflamed (dysfunctional) adipose tissue. The putative underlying mechanisms that link NAFLD and cardiac dysfunction probably have their origin in expanded and inflamed visceral adipose tissue, which releases a variety of pro-inflammatory adipokines, hormones, free fatty acids and other soluble molecules that are potentially involved in the development of systemic insulin resistance and may adversely affect cardiac function and structure. In this complex situation, the liver may function both as the target organ of the resulting systemic abnormalities (induced by expanded visceral adipose tissue) and the source of several pathogenic mediators that may amplify the cardiac and vascular damage. Indeed, NAFLD, especially its necro-inflammatory variant, may exacerbate insulin resistance and releases a myriad of pro-inflammatory factors and vasoactive and thrombogenic molecules that play important roles in the development and progression of LVDD and structural cardiac diseases [1,6–9,39]. Preliminary evidence also suggests that patients with NAFLD have changes in cardiac substrate metabolism that may produce myocardial functional and structural abnormalities. For example, Rijzewijk *et al*. [40] found that uncomplicated type 2 diabetic male patients with higher intra-hepatic triglyceride content on proton magnetic resonance spectroscopy had higher myocardial insulin resistance, lower myocardial high-energy phosphate metabolism (as measured by the phosphocreatine/adenosine triphosphate ratio) and lower myocardial perfusion compared with their counterparts with lower intra-hepatic triglyceride content; notably, these abnormalities in myocardial substrate metabolism were more severe among those with higher intra-hepatic triglyceride content even after adjustment for potential confounders [40]. Emerging evidence also suggests that the coexistence of obesity-related increases in fat accumulation in the myocardium/pericardium might additionally exert local adverse effects that result in functional and structural cardiac alterations [9,38,39]. Rijzewijk *et al*. [41] found that myocardial steatosis was much higher in uncomplicated type 2 diabetic male patients with preserved systolic function than in age- and BMI-matched healthy controls, and that higher intra-myocardial triglyceride content was associated with LVDD, independently of age, BMI, visceral adipose tissue, heart rate and blood pressure. However, in a recent elegant study assessing the effect of different ectopic fat depots on LV function in 75 non-diabetic men with NAFLD, Granér *et al*. reported that only intra-hepatic triglyceride content and visceral adipose tissue were independent predictors of LV diastolic function, whereas myocardial triglyceride content, epicardial and pericardial fat were not associated with diastolic function measures [42]. This further supports the possibility that the association of NAFLD with LVDD may be because of toxic systemic effects. However, further research is needed to better elucidate these issues.

Our study has some important limitations. Firstly, the cross-sectional design of the study limits our ability to establish both the temporality and the causality of the observed associations. Secondly, the diagnosis of NAFLD was based on ultrasound imaging and the exclusion of other known etiological factors of chronic liver diseases, but was not confirmed by liver biopsy. Although some non-differential misclassification of NAFLD on the basis of ultrasonography is likely (*i*.*e*., some of our diabetic control patients could have underlying NAFLD despite fairly normal serum liver enzymes and negative ultrasonography examination), this limitation would serve to attenuate the magnitude of our effect measures toward null; thus, our data can probably be considered conservative estimates of the relationship between NAFLD and LVDD. Finally, because our sample comprised white type 2 diabetic individuals who were followed at an outpatient clinic, our results may not necessarily be generalizable to other non-white diabetic populations.

Notwithstanding these limitations, our study has important strengths, including the large sample size, the use of tissue Doppler imaging (with speckle-tracking strain measurements), which is the most practical and reproducible method for diagnosing early LVDD, the completeness of the dataset, the ability to adjust for multiple risk factors and potential confounders and the exclusion of patients with cirrhosis; we believe that the inclusion of patients with such complication would possibly have confounded the interpretation of the data.

In conclusion, these results indicate that NAFLD is independently associated with echocardiographic features of early LVDD in type 2 diabetic patients with preserved systolic function and without overt IHD. Further studies are needed to explore whether improvement in NAFLD (or future treatments for NAFLD) will ultimately delay or prevent the development of LVDD in patients with type 2 diabetes mellitus.
